# The expression profile and clinical relevance of miR-125a-5p in individuals with chronic periodontitis

**DOI:** 10.2340/aos.v85.45028

**Published:** 2026-01-16

**Authors:** Senqiang Li, Baoqing Fang, Qian Sun

**Affiliations:** a Department of Stomatology, Tongde Hospital of Zhejiang Province, Hangzhou, Zhejiang, China; b Deparment of Stomatology, Xihu District Hospital of Chinese and West Medicine Hangzhou, Hangzhou, Zhejiang, China; c Department of Nursing, Tongde Hospital of Zhejiang Province, Hangzhou, Zhejiang, China

**Keywords:** Chronic periodontitis, miR-125a-5p, BTG2, oxidative stress, inflammation

## Abstract

**Background:**

Chronic periodontitis is a prevalent inflammatory disease, and miR-125a-5p’s role in its pathogenesis remains unclear.

**Objective:**

We examined the clinical significance and mechanisms of miR-125a-5p in chronic periodontitis by testing two hypotheses: (1) miR-125a-5p expression is altered in chronic periodontitis and correlates with clinical indicators; (2) miR-125a-5p regulates periodontal ligament fibroblast (PDLF) functions by targeting specific genes, contributing to disease pathogenesis.

**Method:**

Pearson correlation analysis assessed the association between miR-125a-5p expression and key clinical indicators. Receiver operating characteristic (ROC) curve analysis evaluated its diagnostic performance in chronic periodontitis. Periodontal ligament fibroblasts were treated with lipopolysaccharide (LPS) to establish an inflammatory model. miR-125a-5p and B-cell translocation gene 2 (BTG2) expression levels were measured by reverse transcription quantitative polymerase chain reaction (RT-qPCR). Cell count kit-8 (CCK-8) assay and enzyme-linked immunosorbent assay (ELISA) assessed miR-125a-5p’s effects on cell proliferation, oxidative stress, and inflammation. The direct interaction between miR-125a-5p and BTG2 was confirmed using a dual-luciferase reporter assay.

**Results:**

The expression level of miR-125a-5p in the gingival crevicular fluid of patients with chronic periodontitis was significantly upregulated and positively correlated with the severity of periodontal tissue damage. In the LPS-induced PDLFs model, miR-125a-5p expression was upregulated, cell proliferation capacity was suppressed, oxidative stress was induced, and levels of inflammation-related factors were increased. Notably, transfection with a miR-125a-5p inhibitor effectively reversed these effects. Moreover, the study confirmed that BTG2 is a direct target gene of miR-125a-5p, and that miR-125a-5p exerts its regulatory effects on cellular functions through targeting BTG2.

**Conclusion:**

miR-125a-5p is potentially associated with chronic periodontitis, and may serve as a therapeutic target.

## Introduction

Chronic periodontitis is a highly prevalent chronic inflammatory disease of the oral cavity affecting populations worldwide [1]. It originates from the dysregulation of oral microbiota, which triggers a pro-inflammatory response involving innate and adaptive immune cells as well as inflammatory mediators [2]. Clinically, the disease presents with local symptoms including gingival redness and swelling, bleeding on probing (BOP), periodontal pocket formation, and tooth mobility, which significantly impair patients’ chewing function and quality of life [3]. Currently, clinical diagnosis depends on parameters such as BOP, probing pocket depth (PPD), and clinical attachment level (CAL), with supplementary support from imaging techniques [4, 5]. However, this conventional diagnostic approach may lead to missed diagnoses and diagnostic errors. Therefore, the development of novel biomarkers for chronic periodontitis is of great clinical significance.

In recent years, microRNAs (miRNAs) have been recognised as key regulators in the initiation, progression, and regression of chronic periodontitis. For example, the dysregulated expression of miR-205 and high-mobility group box 1 (HMGB1) is strongly correlated with the pathogenesis of chronic periodontitis and may serve as potential biomarkers for disease diagnosis and therapeutic monitoring [6]. Silencing of miR-498 suppresses lipopolysaccharide (LPS)-induced apoptosis and inflammatory responses in periodontal ligament cells (PDLCs), thereby identifying it as a novel target for chronic periodontitis intervention [7]. Notably, miR-125a-5p displays aberrant expression profiles in multiple diseases and contributes to pathological processes by modulating the release of inflammatory factors and influencing cellular behaviours [8–10]. Although miR-125a has been found to be upregulated in patients with periodontal disease [11], its precise mechanisms of action in chronic periodontitis remains unclear, providing an important research direction for uncovering the molecular basis of chronic periodontitis and developing innovative therapeutic strategies.

B-cell translocation gene 2 (BTG2), the first identified member of the BTG/Tob protein family, regulates cell proliferation, apoptosis, and growth [12]. Previous studies have primarily focused on its tumour-suppressive function, specifically, its anti-tumour effects mediated via the Ras signalling pathway [13]. In breast cancer, reduced BTG2 expression is significantly associated with increased tumour volume and upregulated cyclin D1 protein expression [14]. Notably, recent research has shown that elevated BTG2 expression antagonises the anti-apoptotic activity of miR-21-5p, whereas miR-21-5p mitigates doxorubicin (DOX)-induced cardiac injury by downregulating BTG2 levels [15]. BTG2 expression is markedly dysregulated in the periodontal tissues of patients with chronic periodontitis [16]. Given that miR-125a-5p is upregulated in periodontal disease [11], further investigation into the interaction mechanism between miR-125a-5p and BTG2 in chronic periodontitis is anticipated to reveal novel molecular pathways underlying periodontal tissue destruction within the inflammatory microenvironment.

Against the above outlined backdrop, this study comprehensively analysed the expression profiles and regulatory mechanisms of the miR-125a-5p/BTG2 axis in chronic periodontitis. Through the examination of clinical samples and the conduct of *in vitro* functional experiments, this research aims to provide novel insights that may facilitate the early diagnosis of chronic periodontitis and the development of molecular-targeted treatment strategies for the disease.

## Materials and methods

### Research participants

Between January 2022 and May 2024, a total of 96 patients diagnosed with chronic periodontitis and 72 healthy control subjects were recruited from Tongde Hospital of Zhejiang province, China.

Clinical examinations were performed by two trained and calibrated examiners. Prior to the formal examination, a calibration test was conducted by the two examiners on 10 randomly selected participants. The kappa coefficient for the consistency of PPD, CAL, plaque index (PI), and bleeding index (BI) was > 0.8, indicating good inter-examiner consistency. The specific clinical examination parameters and measurement methods are detailed as follows: (1) PPD£ºMeasured using a periodontal probe, with values recorded to the nearest 0.5 mm. Measurements were taken at six sites per tooth, including the mesio-buccal, mid-buccal, disto-buccal, mesio-lingual, mid-lingual, and disto-lingual sites; (2) CAL£ºDefined as the distance from the cementoenamel junction to the base of the periodontal pocket. It was measured at the same six sites as PPD, with values recorded to the nearest 0.5 mm; (3) PI£ºEvaluated using the Silness-Loe PI, with scores ranging from 0 to 3. The scoring criteria were as follows: 0 = no plaque present; 1 = thin plaque film visible only after running a probe over the tooth surface; 2 = moderate plaque visible to the naked eye; 3 = heavy plaque accumulation; (4) BI£ºAssessed using the Loe-Silness BI, with scores ranging from 0 to 3. The scoring criteria were as follows: 0 = no bleeding observed after probing; 1 = slight bleeding visible within 10 s post-probing; 2 = obvious bleeding; 3 = severe bleeding with blood pooling. In addition, all participants underwent dental X-ray examinations, including periapical radiographs and panoramic radiographs, to assess alveolar bone resorption. This radiological assessment served as an auxiliary indicator for the diagnosis of chronic periodontitis.

The inclusion criteria for chronic periodontitis were established in accordance with the *2017 World Workshop on the Classification of Periodontal and Peri-Implant Diseases and Conditions* [17], which are as follows: (1) At least two non-adjacent teeth show ≥1 mm CAL, and at least one tooth has a PPD ≥ 4 mm; (2) At least one site shows BOP; (3) Radiographic imaging reveals alveolar bone loss consistent with chronic periodontitis; (4) The disease progresses slowly, with no clinical or radiographic evidence of aggressive periodontitis.

The inclusion criteria for the healthy control group were as follows: (1) All teeth have PPD ≤ 3 mm, with no CAL and no BOP; (2) Gingiva shows no signs of inflammation (e.g. redness, swelling, hyperplasia); (3) Radiographic imaging demonstrates no alveolar bone resorption, and the participants have no history of gingivitis or periodontal treatment within the past year; (4) No oral mucosal diseases are present, and dental caries are either absent or limited to shallow lesions without pulp or periapical involvement; (5) No systemic diseases (e.g. diabetes, cardiovascular disease), immune disorders, or use of medications that affect oral health.

The exclusion criteria for patients were as follows: (1) Receipt of antibiotic therapy or periodontal treatment within the past 3 months; (2) Presence of immunological disorders, infectious diseases, or other systemic diseases; (3) Being in a pregnant or lactating state.

Clinical data were collected from all participants, including gender, age, PPD, CAL, PI, and BI. This study was approved by the Medical Ethics Committee of Tongde Hospital of Zhejiang province, and all participants provided written informed consent prior to enrolment.

### Sample collection

The collection of gingival crevicular fluid (GCF) samples was conducted in accordance with previously established protocols [18]. Prior to sample collection, participants were asked to refrain from consuming food, beverages, or tobacco products for a minimum of 1 h. They were also required to rinse their mouths with clean water to remove any oral debris. The gingival surface was gently cleaned using a sterile cotton ball to remove plaque. A filter paper strip (approximately 2 mm in width and 5 mm in length) was inserted into the gingival sulcus to a depth of 1–3 mm near the cementoenamel junction with the precaution of ensuring no bleeding occurred. The strip was left in place for 30 s to 2 min, before being carefully removed using sterile forceps. Immediately after removal, the strip was transferred to an Eppendorf (EP) tube and sealed.

### Cell culture and induction

Human PDLFs were obtained from the National Center for the Preservation of Certified Cell Cultures (Shanghai, China). The cells were cultured in Dulbecco’s Modified Eagle Medium (DMEM) supplemented with 10% foetal bovine serum (FBS), 100 U/mL penicillin, and 100 μg/mL streptomycin. The cells were maintained in a humidified incubator with a controlled environment of 37°C and 5% CO₂, and were passaged every 2–3 days.

To simulate the inflammatory microenvironment of chronic periodontitis, PDLFs were treated with 1 μg/mL LPS (93572-42, Sigma) to induce an inflammatory response, thereby establishing an *in vitro* inflammatory model. Meanwhile, PDLFs that received no treatment were designated as the negative control group which was used for comparative analysis of cellular changes between the inflammatory model and the control.

### Cell transfection

Twenty-four hours after LPS treatment, PDLFs were transfected using the Lipofectamine™ 3000 kit (L3000015, Invitrogen). The transfection mixture included miR-125b-5p mimic (50 nM), miR-125b-5p inhibitor (50 nM), and their corresponding negative control groups, which were synthesised by Reebok Bio. After transfection, the cells were further cultured for 24 h in a 5% CO₂ incubator at 37°C. Subsequently, the cells were harvested via trypsin digestion, followed by centrifugation, and then stored at -80°C for subsequent experiments and analysis.

#### Cell count kit--8 assay for cell viability detection

At 0, 24, 48, and 72 h post-incubation, PDLFs were treated with the CCK-8 reagent (40203ES76, YEASEN). After each CCK-8 treatment, the cells were further incubated for 60 min. Subsequently, the absorbance at 450 nm was measured for each well using a microplate reader, and the data were used to assess the proliferative capacity of the cells. Each group was set with 3 replicate wells, and the experiment was independently repeated 6 times.

### Evaluation of oxidative stress and inflammatory markers

The concentrations of malondialdehyde (MDA), superoxide dismutase (SOD), reactive oxygen species (ROS), tumour necrosis factor-α (TNF-α), interleukin-6 (IL-6), interleukin-1β (IL-1β), and interleukin-17 (IL-17) in PDLFs were measured using enzyme-linked immunosorbent assay (ELISA) kits. All ELISA kits were purchased from Invitrogen, and the assays were performed strictly in accordance with the manufacturer’s instructions to ensure the precision and reliability of the experimental data. For each indicator, three replicate samples were used for detection, and the experiment was repeated independently six times.

### Target gene prediction

The online databases miRDB (https://mirdb.org/), miRTarBase (https://mirtarbase.cuhk.edu.cn/~miRTarBase/miRTarBase_2025/php/), and TargetScan (https://www.targetscan.org/vert_80/) were used to predict the target genes of miR-125a-5p. The overlapping predicted target genes were analysed using the JVenn software [19]. In addition, the binding sites between miR-125a-5p and its potential target genes were further predicted using the TargetScan database.

### Dual-luciferase reporter assay

The BTG2 wild-type (BTG2-WT) and BTG2 mutant (BTG2-MUT) reporter plasmids, synthesised by Sangon Biotech (Shanghai, China), were co-transfected into PDLFs in accordance with the manufacturer’s protocol for Lipofectamine 3000 (L3000015, Invitrogen). The transfections included either miR-125a-5p mimic, miR-125a-5p inhibitor, or their corresponding negative controls. At 48 h post-transfection, luciferase activity was measured using the Dual Luciferase Reporter Gene Assay Kit (11402ES60, YEASEN). Renilla luciferase was used as an internal control to verify the direct binding and regulatory relationship between miR-125a-5p and BTG2. Each transfection group was set with three replicate wells, and the experiment was repeated independently six times.

### Total RNA extraction and qPCR

Gingival Sulcus RNA Extraction and RT-qPCR: For gingival sulcus samples, phosphate-buffered saline (PBS) was added to EP tubes containing filter strips, followed by gentle shaking for 10 min to elute RNA from the samples. Total RNA was extracted from the supernatants using the miRcute miRNA Extraction and Isolation Kit (DP501, TIANGEN). cDNA was synthesised from the isolated miRNA using the miRNA 1st Strand cDNA Synthesis Kit (stem-and-loop, MR101, Vazyme), and quantitative analysis was performed using the miRNA Unimodal SYBR qPCR Master Mix (MQ102-01, Vazyme), with U6 small nuclear RNA (U6 snRNA) serving as an internal reference gene.

PDLFs RNA Extraction and RT-qPCR: For PDLFs, RNA extraction was carried out in two separate steps: (1) miRNA was extracted using the MolPure^®^ Cell/Tissue miRNA Kit (19331ES50, YEASEN), and the subsequent reverse transcription and qPCR procedures were identical to those used for gingival sulcus samples; (2) total RNA was extracted using the MolPure^®^ 5min Flash Kit (19231ES50, YEASEN), and the following reverse transcription and qPCR steps were also consistent with the gingival sulcus protocol. Reverse transcription of total RNA was conducted using HiScript III RT SuperMix for qPCR (+gDNA wiper, R323, Vazyme), and GAPDH was used as an internal reference gene. Quantitative PCR was performed using Universal SYBR qPCR Master Mix (Q711-02, Vazyme).

### Statistical analysis

Pearson’s correlation analysis was performed to examine the relationship between miR-125a-5p expression levels and clinicopathological features. The diagnostic value of miR-125a-5p for chronic periodontitis was evaluated using receiver operating characteristic (ROC) curve analysis. All statistical analyses were performed using Statistical Package for the Social Sciences (SPSS) 22.0, while GraphPad Prism was employed for graphical representation. Differences between two independent groups were assessed using an independent-samples t-test, while one-way analysis of variance (ANOVA) was applied for comparisons among multiple groups. The chi-square test was used to analyse categorical data, and post hoc pairwise comparisons were performed using Tukey’s test. All data in the figures are presented as the mean ± standard deviation (SD), with error bars representing the SD. Statistical significance was defined as a *p* < 0.05.

## Results

### Analysis of differences in clinical baseline characteristics and chronic periodontitis-related indicators between groups

An *a priori* power analysis was conducted using G*Power 3.1 software with an effect size of 0.5, an alpha error probability of 0.05, and a desired power (1 – beta error probability) of 0.8, yielding a minimum required sample size of 64 per group. A post hoc power analysis was subsequently performed with the same effect size and alpha level, along with the actual sample sizes of 96 cases and 72 controls, which resulted in an achieved power of 0.89. These results indicate that the sample size in the chronic periodontitis group (*n* = 96) and the healthy control group (*n* = 72) is sufficient to detect the expected differences with adequate statistical power.

To clarify the differences in clinical characteristics between patients with chronic periodontitis and healthy individuals, we compared baseline demographic data and key periodontal parameters between the two groups. A healthy control group (HG, *n* = 72) and a chronic periodontitis group (CP, *n* = 96) were enrolled in this study. No statistically significant differences were found between the two groups with respect to age (*t* = 1.321, *p* = 0.19), gender distribution (χ² = 0.098, *p* = 0.75), or smoking status (χ² = 0.089, *p* = 0.93) (Table 1). The CP group showed significant higher levels in PPD (*t* = 17.91, *p* < 0.001), CAL (*t* = 19.31, *p* < 0.001), PI (*t* = 23.98, *p* < 0.001), and BI (*t* = 22.39, *p* < 0.001, Table 1).

**Table 1:** Comparison of clinical data between the HG group and CP group.

Statistical indicators	HG (*n* = 72)	CP (*n* = 96)	Statistic	*P*-value
Age	50.43±4.76	49.56±3.76	*t* = 1.321	0.190
Gender (Male/Female)	40/32	51/45	χ² = 0.098	0.750
Probing pocket depth (mm)	1.83±0.75	5.35±1.54	*t* = 17.910	< 0.001
Clinical attachment loss (mm)	0.75±0.15	5.98±2.29	*t* = 19.310	< 0.001
Plaque index	0.54±0.50	2.40±0.49	*t* = 23.980	< 0.001
Bleeding index	1.01±0.85	3.83±0.78	*t* = 22.390	< 0.001
Smoking status (N/Y)	37/35	50/46	χ² = 0.089	0.930

### Expression of miR-125a-5p in chronic periodontitis and its correlation with clinical indicators

**Figure 1:**
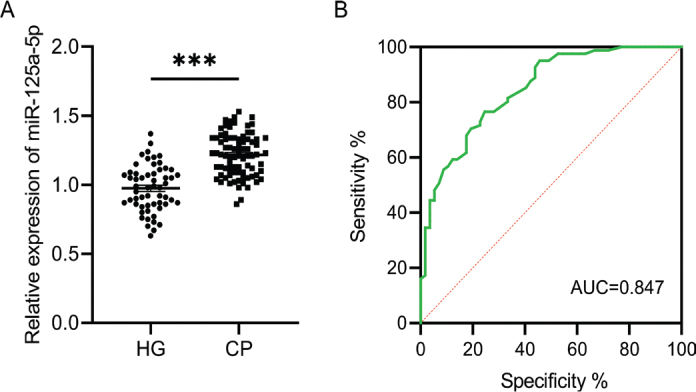
miR-125a-5p is elevated in individuals with chronic periodontitis and exhibits potential as a diagnostic indicator. (A) miR-125a-5p expression levels in GCF samples from healthy individuals and patients with chronic periodontitis were measured using qPCR; (B) ROC curve analysis was conducted to assess the diagnostic accuracy, and the optimal cut-off value was determined as 1.09, with a corresponding Youden index of 0.519, *** *p* < 0.001.

Based on the differences in clinical indicators between the groups, we further analysed the expression characteristics of miR-125a-5p in GCF and its association with periodontitis in the two subject groups. The relative expression of miR-125a-5p in GCF was significantly higher in the CP group compared to the HG group (*t* = 8.72, *p* < 0.001, Figure 1A). Receiver operating characteristic curve analysis revealed that miR-125a-5p had an area under the curve (AUC) of 0.847 (95% CI: 0.783–0.911), with a sensitivity of 76.5% and specificity of 71.9%, for identifying chronic periodontitis (Figure 1B). Pearson correlation analysis indicated a positive association between miR-125a-5p expression and PPD (*r* = 0.898, 95% CI: 0.851–0.931, *p* < 0.001), CAL (*r* = 0.894, 95% CI: 0.845–0.928, *p* < 0.001), PI (*r* = 0.769, 95% CI: 0.672–0.840, *p* < 0.001), and BI (*r* = 0.866, 95% CI: 0.806–0.909, *p* < 0.001, Table 2).

**Table 2:** Pearson correlation analysis was performed to investigate the correlations between miR-125a-5p and various clinical indicators in patients.

Indicators	Pearson correlation coefficient	*P-*value
Probing pocket depth (mm)	0.898	< 0.001***
Attachment loss (mm)	0.894	< 0.001***
Plaque index	0.769	< 0.001***
Bleeding index	0.866	< 0.001***

### miR-125a-5p expression is increased in LPS-stimulated PDLFs, and it suppresses cell proliferation and viability

**Figure 2:**
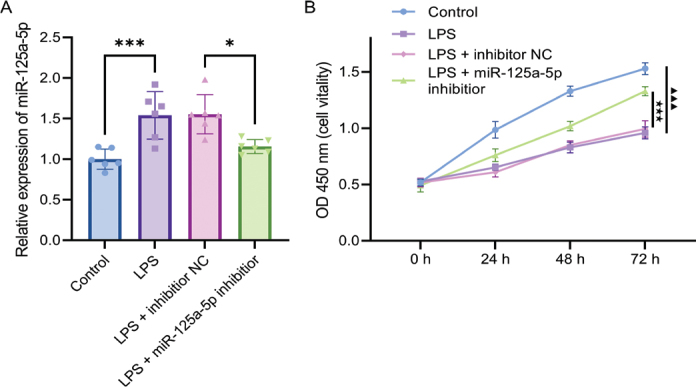
miR-125a-5p is increased in LPS-stimulated PDLFs and inhibits cell proliferation. (A) qPCR analysis was performed to measure miR-125a-5p expression levels in the LPS-induced PDLC model, *** *p* < 0.001; (B) Cell proliferation was evaluated using the CCK-8 assay. The difference between the Control and LPS groups is marked by ▲, ▲▲▲ *p* < 0.001; the comparison between the LPS group and the LPS + miR-125a-5p inhibitor group is indicated by ★, ★★★ *p* < 0.001. *n* = 6 independent experiments. All data are presented as mean ± standard deviation.

To investigate the functional role of miR-125a-5p in the pathological microenvironment of periodontitis, we first established an *in vitro* inflammatory model by stimulating PDLFs with LPS. We then analysed the expression changes of miR-125a-5p and its regulatory effect on cell proliferation. After PDLFs were treated with LPS for 24 h, the miR-125a-5p expression level was significantly higher than that in the control group. Furthermore, transfection with the miR-125a-5p inhibitor led to a significant decrease in its expression (*p* < 0.001, Figure 2A).

Following 72 h of exposure to LPS, the proliferative activity of PDLFs was significantly lower than that in the control group. Moreover, when miR-125a-5p expression was downregulated using an inhibitor, cell viability was significantly restored (*p* < 0.001, Figure 2B).

### miR-125a-5p induces oxidative stress and promotes inflammatory responses in PDLFs

**Figure 3:**
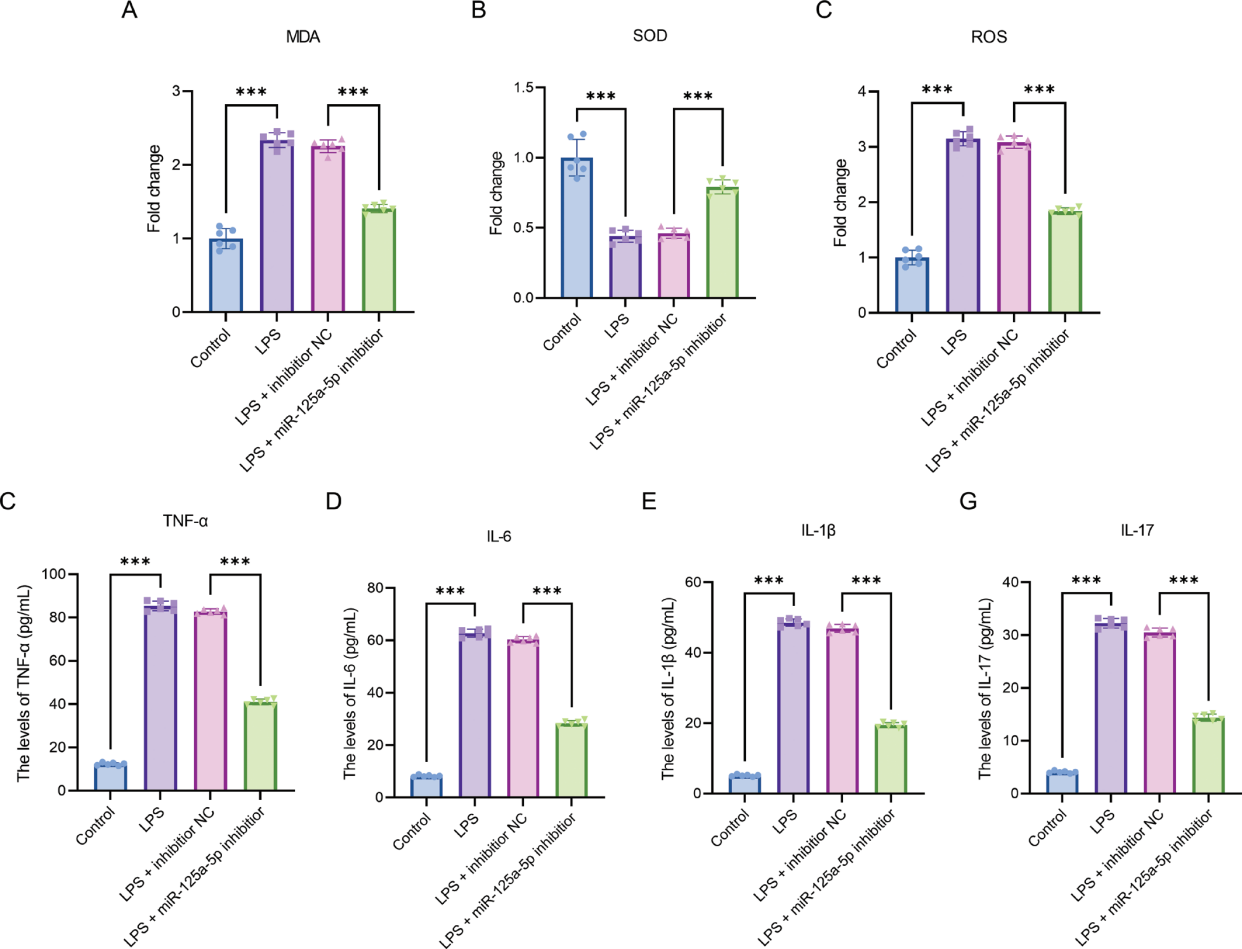
miR-125a-5p triggers oxidative stress and enhances inflammatory reactions in PDLFs. (A) MDA levels in PDLFs were quantified using ELISA; (B) SOD concentrations were assessed by ELISA; (C) ROS levels were determined through ELISA; (D) TNF-α expression was measured via ELISA; (E) IL-6 levels were evaluated using ELISA; (F) IL-1β concentrations were analysed by ELISA; (G) IL-17 levels in PDLFs were detected via ELISA. ***p < 0.001. n = 6 independent experiments. All data are presented as mean ± standard deviation.

Based on the expression changes of miR-125a-5p in the LPS-induced inflammatory model, we conducted further inhibitor intervention experiments to analyse its regulatory effects on oxidative stress and inflammatory responses in PDLFs. Lipopolysaccharide administration led to a significant increase in intracellular MDA and ROS levels, along with a significant decrease in SOD activity (*p* < 0.001). Silencing of miR-125a-5p reversed these effects, as evidenced by reduced MDA and ROS levels and a recovery of SOD activity to 0.79 ± 0.08-fold compared to the LPS group (*p* < 0.001, Figure 3A–C).

Lipopolysaccharide treatment also led to a significant increase in the levels of TNF-α, IL-6, IL-1β, and IL-17. Nevertheless, following the inhibition of miR-125a-5p, the expression of these inflammatory cytokines decreased significantly (*p* < 0.001, Figure 3D–G).

### BTG2 is a target gene of miR-125a-5p

**Figure 4:**
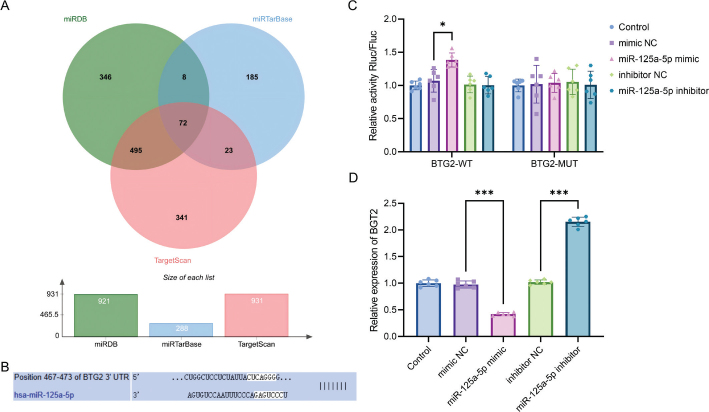
BTG2 is directly regulated by miR-125a-5p. (A) A Venn diagram depicting the overlapping predicted target genes of miR-125a-5p from TargetScan, miRDB, and miRTarBase; (B) Putative binding sites between miR-125a-5p and BTG2 were predicted using TargetScan; (C) Confirmation of the binding relationship between miR-125a-5p and BTG2 using a dual-luciferase reporter system; (D) BTG2 expression levels were assessed using qRT-PCR. * *p* < 0.05; *** *p* < 0.001. *n* = 6 independent experiments. All data are presented as mean ± standard deviation.

To elucidate the molecular mechanism underlying miR-125a-5p–mediated regulation of PDLF functions, we integrated bioinformatics predictions with *in vitro* experiments to validate potential target genes and their regulatory relationships. The target genes of miR-125a-5p were predicted using the TargetScan, miRDB, and miRTarBase databases. Following Venn analysis, 72 potential target genes were identified, among which BTG2 exhibited a relatively high score (Figure 4A). Further analysis with TargetScan revealed that BTG2 and miR-125a-5p potentially share binding sites (Figure 4B).

Dual-luciferase reporter assays demonstrated that overexpression of miR-125a-5p led to a significant decrease in the luciferase activity of BTG2-WT (*p* < 0.001), while no significant change was observed in the BTG2-MUT group (Figure 4C). In LPS-treated PDLFs, overexpression of miR-125a-5p led to downregulation of BTG2 mRNA expression (*p* < 0.001), whereas silencing of miR-125a-5p resulted in upregulation of BTG2 expression (*p* < 0.001, Figure 4D).

## Discussion

As reported by the World Health Organization, chronic periodontitis affects 35 to 50% of the global population, making it one of the most prevalent chronic inflammatory diseases of the oral cavity [20]. Epidemiological studies have suggested a potential association between chronic periodontitis and an increased risk of oral squamous cell carcinoma, although this relationship is not considered a direct progressive one and is confounded by factors such as smoking, alcohol consumption, and poor oral hygiene [21, 22]. Currently, clinical diagnosis of chronic periodontitis mainly relies on periodontal probing (e.g. PPD, CAL) and radiological evaluation; however, early-stage lesions are often difficult to detect due to the lack of obvious characteristic symptoms, highlighting the need for more sensitive diagnostic tools. Earlier studies have shown that miR-1226 has the potential to function as a diagnostic biomarker for periodontitis and can predict both the progression and severity of the disease [18]. Meanwhile, miR-155 and miR-146a are highly expressed in the saliva of patients with periodontitis and exhibit positive correlations with disease severity and clinical parameters [23]. Therefore, screening for biomarkers in saliva or GCF has emerged as a promising direction for the diagnosis of periodontal disease [24, 25]. Numerous studies have demonstrated that miR-125a-5p can serve as a diagnostic biomarker for a range of diseases, including early gastric cancer, rheumatoid arthritis, and salivary gland adenoid cystic carcinoma [26–28]. Our findings revealed that the expression level of miR-125a-5p in the GCF of individuals with chronic periodontitis was markedly elevated compared to that in healthy subjects, which aligns with previous studies [11]. The expression level of miR-125a-5p is significantly correlated with key clinical indicators, including PPD and BI, indicating its potential clinical relevance that is comparable to these established parameters. In the future, miR-125a-5p could be integrated with clinical parameters to aid in the diagnosis of chronic periodontitis, thereby enhancing diagnostic comprehensiveness and accuracy through the combined use of multiple biomarkers.

Oxidative stress and inflammation are central mechanisms in the pathological process of chronic periodontitis, and their imbalance can significantly exacerbate the destruction of periodontal tissues [29]. Oxidative stress is a pathological state characterised by an imbalance between oxidative processes and antioxidant defenses within the body, which leads to tissue damage through excessive production of ROS [30]. Multiple studies have shown that levels of oxidative markers such as MDA and ROS in GCF and serum are significantly elevated, while antioxidant enzymes such as SOD are reduced in patients with chronic periodontitis [31, 32]. Meanwhile, in the local periodontal microenvironment, the balance of the pro-inflammatory cytokine network is disrupted, resulting in the excessive production of IL-1β, TNF-α, IL-6, and IL-17, which further amplifies the inflammatory cascade [2, 33]. In the present study, the LPS-induced PDLFs model exhibited a typical oxidative-inflammatory imbalance, with increased MDA and ROS levels, decreased SOD activity, and elevated TNF-α and IL-6 after LPS treatment, which is highly consistent with the pathological features reported in previous literature [34]. Notably, transfection with a miR-125a-5p inhibitor significantly improved intracellular oxidative stress indicators and reduced the release of pro-inflammatory factors. These results suggest that miR-125a-5p may play a pro-pathogenic role in the inflammatory microenvironment of chronic periodontitis by regulating the oxidative stress-inflammatory axis. It is important to note that in this study, the miR-125a-5p inhibitor only partially reversed the LPS-induced functional abnormalities in PDLFs, and oxidative stress markers as well as inflammatory factors did not return to levels observed in the healthy control group. These findings indicate that miR-125a-5p is not the sole mediator of periodontal inflammation and tissue damage. Rather, the LPS-triggered pathological process involves the coordinated activity of multiple signalling pathways, such as TLR4/NF-κB and p38 MAPK, and regulatory molecules, including other miRNAs and cytokines [35, 36]. An abnormally high expression of miR-125a-5p may exacerbate oxidative damage and inflammatory infiltration in periodontal tissues by targeting and regulating the transcription of antioxidant genes and inflammation-related genes. This finding provides a new direction for clinical intervention by inhibiting miR-125a-5p expression, potentially breaking the oxidative-inflammatory vicious cycle and opening up a new pathway for molecularly-targeted treatment of chronic periodontitis.

In the present study, bioinformatics prediction combined with dual-luciferase reporter gene assays confirmed that BTG2 is a direct target gene of miR-125a-5p. As a key member of the BTG/Tob protein family, BTG2 participates in the regulation of cell proliferation, apoptosis, and development by modulating transcriptional and translational activities [37]. Aberrant expression of BTG2 has been associated with multiple disorders, including tumorigenesis and diabetic nephropathy [12]. Notably, the expression level of BTG2 in the periodontal tissues of patients with chronic periodontitis is significantly altered, suggesting that BTG2 may be involved in the inflammatory signalling network to regulate the destruction of periodontal tissues [16]. Functional experiments revealed that elevated miR-125a-5p expression reduced BTG2 mRNA expression in PDLFs, whereas suppression of miR-125a-5p increased BTG2 expression. These results confirm that miR-125a-5p inhibits BTG2 expression by binding to the 3’-UTR of BTG2 mRNA. Collectively, the miR-125a-5p/BTG2 pathway may serve as a key regulatory mechanism in the pathogenesis of chronic periodontitis, providing a promising molecular target for the development of innovative therapeutic strategies.

While the present study elucidated the expression pattern of miR-125a-5p in chronic periodontitis and its interaction mechanism with BTG2, several limitations should be acknowledged. Firstly, the current study had a small sample size; thus, additional research with larger patient cohorts is required to validate the applicability of miR-125a-5p as a diagnostic marker for chronic periodontitis. Secondly, miR-125a-5p may regulate other target genes involved in the pathogenesis of chronic periodontitis, therefore, future studies should focus on comprehensively exploring its downstream regulatory network. This cross-sectional study confirmed the differential expression of miR-125a-5p between diseased and healthy states; but failed to capture the dynamic changes in this microRNA during disease initiation, progression, or treatment. Consequently, the clinical utility of miR-125a-5p as a biomarker for chronic periodontitis requires further investigation in subsequent studies.

In conclusion, the present study demonstrates that miR-125a-5p is markedly overexpressed in chronic periodontitis and promotes the degradation of periodontal tissues by enhancing oxidative stress and inflammatory responses in PDLFs through targeted inhibition of BTG2. Importantly, the miR-125a-5p/BTG2 regulatory pathway provides a novel target and approach for the development of molecularly targeted therapies for chronic periodontitis.

## Data Availability

The datasets used and/or analysed during the current study are available from the corresponding author on reasonable request.
